# Supplementary material for: Effects of Individual Amino Acids on PPARα Transactivation, mTORC1 Activation, ApoA-I Transcription and pro-ApoA-I Secretion
Source: Int J Mol Sci. 2022 May 28;23(11):6071. doi: 10.3390/ijms23116071 (PMC9181357; doi:10.3390/ijms23116071)
Supplement: Supplementary file 1 [file ijms-23-06071-s001.zip › ijms-1598922-supplementary.pdf]

Supplementary Table S1. The chemical characteristics of different amino acids that were selected in this study.

**Amino acid**	**Chemical characteristics**
Glutamine	Polar, hydrophilic
Leucine	Non-polar, hydrophobic
Proline	Non-polar, neutral
Histidine	Polar, hydrophilic
Glutamic acid	Polar, Acidic
Tryptophan	Non-polar, hydrophobic

Supplementary Table S2. Amino acid concentrations in different medium compositions.

	**Amino acid concentration (mM)**					
	Glutamine	Leucine	Proline	Histidine	Glutamic acid	Tryptophan
MEM	2,00	0,40	0,00	0,20	0,00	0,05
FBS	0,00	2,97	1,68	0,79	2,25	0,26
MEM +10% FBS	1,80	0,65	0,17	0,26	0,23	0,07
Times-fold +10% FBS	0,90	1,63	-	1,30	-	1,40
